# Baseline Gross Motor Function Affects the Outcome of Robot-Assisted Therapy in Ambulatory Individuals with Spastic Cerebral Palsy

**DOI:** 10.3390/brainsci11121563

**Published:** 2021-11-26

**Authors:** Faustyna Manikowska, Anna Krzyżańska, Paweł Chmara, Brian Po-Jung Chen, Marek Jóźwiak

**Affiliations:** 1 Gait and Motion Analysis Laboratory, Department of Pediatric Orthopedics and Traumatology, Poznań University of Medical Sciences, 61-545 Poznań, Poland; chmara.pawel@gmail.com (P.C.); jozwiakmp@gmail.com (M.J.); 2 X-Rehab, Poznań Rehabilitation Center, 61-545 Poznań, Poland; gera123@poczta.onet.pl; 3 Department of Pediatric Orthopedics, Chang Gung Memorial Hospital, Linkou, Taoyuan 33305, Taiwan; brianchen.md@icloud.com

**Keywords:** cerebral palsy, robot-assisted therapy, gross motor function

## Abstract

Robotic-assisted therapy (RAT) is a task-specific approach for treating gait disorders in individuals with neurological impairments. However, the effectiveness of RAT is not clear for different severities of involvement, pathologies, and ages. This study aimed to assess the functional and clinical status outcomes after RAT in individuals with cerebral palsy (CP). Twenty-eight individuals with bilateral spastic CP were enrolled (female = 10; male = 18; age = 15.2 ± 2.0 years). The RAT program consisted of 30 sessions: five sessions weekly for six weeks. Gross Motor Function Measure (GMFM) and clinical physical examinations were evaluated before and after RAT. Our results suggested that the RAT program with the described protocol can improve the general gross motor functions of individuals with CP in Gross Motor Function Classification System (GMFCS) levels I and II, and primarily improves performance on less complex GMFM items for those in GMFCS levels III and IV. The lower baseline functional level was related to a greater functional improvement. Older individuals were noticed to improve more in GMFM dimension D. Regarding impairments evaluated by clinical examinations, no change was found after RAT intervention. It is worth mentioning that the strength of knee muscles was not affected either.

## 1. Introduction

Cerebral palsy (CP) is a non-progressive damage of the immature brain, which manifests by complex impairment. Weakness, reduced selective motor control, and spasticity, among other symptoms, affect the motor function ability of patients. The heterogeneity of this disease being caused by injury to the brain’s motor system leads to infinite combinations of clinical symptoms. Consequently, individuals with neurological deficits present various degrees of disability that interfere with their function and daily life activities [1].

The primary treatment goal for children with CP is to facilitate the individual’s ability to perform activities in daily life [2,3]. The relationship between motor impairment, function, and clinical status has a fundamental impact on therapy outcomes. Choosing the appropriate therapy for these individuals is very challenging. Treatments must be based on each individual’s needs, abilities, and limitations. Treatment goals should meet subjective aims and at the same time take into consideration the restrictions resulting from neurological impairments, functional deficits, and body deformities.

Robotic-assisted therapy (RAT) is a relatively new method of treatment for patients with neurological impairments originally developed and applied as an advanced task-specific approach for treating gait disorders. The advantages of RAT are the ability to adjust therapy to individual requirements and to modify therapy programs to fit each individual’s demands [4]. Robot-assisted gait training and computer-assisted systems allow different degrees of body weight support, maintain the appropriate alignment of body segments, and provide movement guidance to the joints [5,6]. It was also proven that RAT has a positive impact on some functional aspects, including walking and standing ability and muscle strength [1,7], and gait training can be conducted more effectively than conventional therapy [3,8]. However, the effectiveness of RAT is not clear for different severities of involvement, pathologies, and ages [9,10,11].

Previous studies focused mainly on functional changes after RAT [11,12,13,14,15,16] and were based on a wide range of outcome measures, equipment, and patient profiles [6,9,11,17]. We are not aware of any study which investigated changes in presentations evaluated by clinical examinations after individuals with CP were treated by RAT. Moreover, it is not clear which groups of individuals may benefit the most from this type of therapy. The impact of the pre-interventional condition of an individual on the final effects of therapy is not clear either.

The primary aim of the study was to assess the changes in the functional and clinical status of patients with CP after RAT. The secondary aim was to assess the relationship between the baseline condition of an individual and the short-term outcomes of RAT. To achieve the aims, changes in function were assessed with the Gross Motor Function Measure (GMFM); clinical status was assessed with clinical examination consisting of passive joint range-of-motion (ROM), spasticity, and selectivity tests; and muscle strength was assessed both manually and instrumentally.

## 2. Materials and Methods

### 2.1. Participants

In total, 28 individuals with bilateral spastic CP were enrolled (female, *N* = 10; male, *N* = 18; age = 15.2 ± 2.0 years; range: 11.9–18.8 years, height: 162.7 ± 8.6, weight: 55.6 ± 8.8).

Patients were recruited from the Technology-Based Therapy Department of the local rehabilitation hospital. Inclusion criteria were: (1) ability to apply exoskeleton: 150 cm or more, no severe lower limb joint contracture preventing its use (knee flexion contraction no more than 12°, hip joint range of motion at least 60° of flexion and 5° of extension); (2) ability to follow oral instructions; (3) vision and mental health allow understanding virtual reality tasks; and (4) functional level enabling RAT (GMFCS level I–IV). Exclusion criterion was: received surgery or botulinum toxin injection within the last 6 months before the examination. Participants were divided into two groups based on their ambulatory function classified by GMFCS: group 1—independent ambulators (GMFCS level I, *N* = 11; level II, *N* = 8; sex: female, *N* = 7; male, *N* = 12; age = 15.2 ± 2.0 years; age range: 11.9–18.8 years, height: 164.5 ± 8.6 cm, weight: 55.9 ± 9.2 kg); group 2—dependent ambulators with assistive devices (GMFCS level III, *N* = 5; level IV, *N* = 4; sex: female, *N* = 3; male, *N* = 6; ag e= 15.2 ± 2.0 years; age range: 11.9–18.8 years; height: 158.8 ± 8.6 cm; weight: 54.8 ± 8.5 kg).

The appropriate Institutional Review Board approved the study. Written consent was acquired from all participants. For participants under the age of 18, consent was obtained from a parent or legal guardian.

### 2.2. Protocols

The therapy program was based on RAT, which consists of 30 sessions: five therapeutic sessions per week for six weeks, with a two-week break every two weeks. The first session was primarily for the assessment procedure, adjustment of the equipment, and determining the endurance and dosage for each individual. Based on data collected from each piece of equipment, the individual baseline for each patient was established. Along with the therapy progress, the dosage, amount of support, and level of difficulty were adjusted for each patient based on feedback and the real-time data collected.

At each session, the participant underwent gait training (with exoskeleton and treadmill) and balance training (on stabilometric and dynamographic platforms) according to the following therapeutic schedule (Table 1):

### 2.3. Assessment Protocol

Patients were evaluated at two time points: before RAT and at the last day of RAT. Each evaluation session consisted of:

#### 2.3.1. Functional Assessment

(a) Gross motor function: the 88-item GMFM (GMFM-88) was tested according to the instruction manual [18].
(b) Ambulatory function: the GMFCS was used to classify individuals with CP into five levels of ambulatory ability, where level I means independent walking with minimal limitation and level V means no ambulation ability.

#### 2.3.2. Clinical Assessments

(a) Strength: manual muscle test (MMT) grading muscle strength from 1 to 5 was used. Standard positions and procedures for MMT were applied for the following muscles: hip flexors, hip extensors, knee flexors, knee extensors, ankle dorsiflexors, and the ankle plantar flexors of each lower limb [19].
(b) Muscle tone impairment: the modified Ashworth scale (MAS) and the Tardieu scale were used to assess the spasticity of hip flexors (HF), rectus femoris (RF), hamstrings (HS), and plantar flexors (PF). Standard examining positions and procedures were applied [20,21].
(c) Selective motor control (SMC): SMC of the hip, knee, and ankle were graded from 2 (completely isolated of movement) to 1 (partially isolated movement) or to 0 (lack of ability to perform isolated movement) [22].
(d) Passive range of motion (ROM): measured with a manual goniometer in standard positions and procedures for hip, knee, and ankle joint of each lower limb in all three anatomical planes; the Thomas test, unilateral and bilateral popliteal angle test, and hip anteversion angle were also measured [19].

#### 2.3.3. Instrumental Strength Assessment

Isometric strength of knee flexors (HS) and knee extensors (RF) was tested using Biodex System (Biodex System 4 Pro; Biodex Medical Systems, Inc., Shirley, NT, USA). Maximal muscle strength was defined as the highest peak torque (Nm). The test was conducted in the standard positions and procedures [23].

### 2.4. Data Analysis

Calculations were performed using Statistica version 13 (TIBCO Software Inc., Palo Alto, CA, USA) and PQStat (PQStat Software, Poznań, Poland). The level of significance was set at α = 0.05. The normality of the distribution of variables was tested using the Shapiro–Wilk test. To test the changes over time, for the continuous parameters, the paired *t*-test (in cases of compliance with the normal distribution at both time points) or the Wilcoxon test (in case of non-compliance with the normal distribution) was calculated. The McNemar or Bowker symmetry tests were calculated for categorical variables. To investigate the relationship between the variables, the Pearson linear correlation or Spearman’s rank correlation coefficients were calculated. The Mann–Whitney test, unpaired *t*-test, or Cochran–Cox test were calculated to compare variables between groups. A post-hoc power analysis was performed on the improvement of outcome measures with the final sample size. 

### 2.5. Outcome Measures

The primary outcome measures were the changes in all assessed parameters calculated as differences between the first and the second examination of each participant. We analyzed the following:

- GMFM-88 total score: achieved percentage of the total possible score;
- Clinical examination: range of motion measured in degrees by manual goniometer;
- Motor impairments (weakness, lack of SMC, spasticity): changes in the severity of symptoms.

## 3. Results

### 3.1. Functional Assessment

We found a statistically significant improvement in gross motor functions assessed by GMFM both in total score (*p* < 0.01) and in each specific dimension (dimension A: *p* < 0.01; dimension B: *p* = 0.02; dimension C: *p* < 0.01; dimension D: *p* < 0.01; dimension E: *p* < 0.01). The same analysis was performed based on the ambulation ability. Individuals in group 1 showed statistically significant changes in GMFM total score (*p* < 0.01) and in all GMFM dimensions (dimension A: *p* = 0.04; dimension B: *p* = 0.03; dimension C: *p* = 0.02; dimension D: *p* < 0.01; dimension E: *p* < 0.01) (Table 2). In group 2, the improvement was shown in the GMFM total score (*p* < 0.01); however, significant changes were observed only in dimension A (*p* = 0.03) and dimension C (*p* = 0.01) (Table 2).

### 3.2. Clinical Assessment

There were significant changes in bilateral popliteal angle (*p* = 0.01) and Thomas test (*p* = 0.04). We didn’t find any significant changes in spasticity and selectivity in any of the examined muscles or joints (Table 3).

### 3.3. Instrumental Strength Assessment

Instrumental assessment of muscle strength did not show significant changes in the strength of the knee flexors (*p* = 0.05) or knee extensor muscles (*p* = 0.46) after RAT (Table 4).

### 3.4. Impact on Improvement

We found that baseline gross motor function, age, and GMFCS level were associated with some changes in function. We found that a lower baseline GMFM total score was associated with a higher improvement of GMFM total score (*p* < 0.01, Rs = −0.59) after RAT. The same relationship was found in dimension A (*p* < 0.01, Rs = −0.75), B (*p* < 0.01, Rs = −0.60), and C (*p* < 0.01, Rs = −0.59). GMFCS level was associated with changes in dimension A (*p* = 0.04). Age was associated with changes in dimension D (*p* = 0.03, Rs = 0.42). 

Comparing the differences in time, the statistical power for GMFM was in the range 97–100%, 37–96% for GMFM dimensions, and 74% for clinical examination. 

## 4. Discussion

The primary aim of the study was to assess the changes in the functional and clinical status of individuals with spastic CP after RAT.

We found statistically significant changes in the functional abilities of patients assessed by GMFM (*p* < 0.01) and in each GMFM dimension (A: *p* < 0.01; B: *p* = 0.02; C: *p* < 0.01; D: *p* < 0.01; E: *p* < 0.01). It is worth emphasizing that those improvements were not similar between groups. While group I (independent walkers) improved in all GMFM dimensions, group 2 (walking with aids) showed changes only in less difficult functions such as lying and rolling (dimension A) or crawling and kneeling (dimension C). Changes in the gross motor are interesting because significant benefits from the RAT were expected mainly in dimensions D and E in the more affected group, but not in lower functions, and especially not for independent walkers.

We noticed improvements in some clinical measurements, for example bilateral popliteal angle and Thomas test. Other parameters tested in the clinical examination did not show changes in range of motion, muscle tone, selective motor control, or muscle strength. Our findings suggested that the effect of RAT on individuals with spastic CP cannot be detected by most clinical examinations.

The instrumental muscle strength assessment around knee joints did not show any significant changes (knee flexors *p* = 0.05; knee extensor muscles *p* = 0.46); however, some changes for the knee flexors were not statistically significant. Our data suggested that RAT might not improve the strength of every knee joint muscle.

The secondary aim of the study was to assess the relationship between the baseline condition of an individual and short-term outcomes to identify subjects who could benefit the most from RAT. Our analysis showed that individuals with lower baseline functional abilities may achieve better results after the intervention of RAT (with average positive correlation). Age has a weak positive correlation with improvement in standing, that is, older patients may achieve greater improvement in the dimension D items of GMFM. Similarly, weak positive correlation was found between GMFCS levels and GMFM dimension A, that is, poor walking ability is associated with greater changes in less difficult functions like lying and rolling.

The majority of previous studies were focused on functional changes after RAT and showed significant GMFM improvement in dimensions D and E [24,25,26,27,28]. Those studies considered CP patients as one group, despite its huge diversity of clinical symptoms. Detailed analysis regarding the ambulatory level of patients did not provide unambiguous results. Hendel et al. showed that significant improvement in GMFM dimensions D and E were found only in GMFCS level IV individuals but not level II or III individuals [29]. In contrast, Borgraffe et al. concluded that greater benefits may be achieved in mildly affected subjects (GMFCS level I and II) in comparison with more affected ones (GMFCS level III and IV) [27]. Our data showed that even though GMFM total score improves in both groups, individuals in GMFCS levels I and II improved in all five dimensions, while more affected patients (level III and IV) improved only in dimensions A and C.

An investigation by Schroeder et al. on the association between GMFM baseline score and response of RAT treatment showed that patients presenting higher motor abilities at baseline had greater improvements after therapy [30]. In addition, they showed a negative relationship between age and dimension D of GMFM. Willoughby reported that the effectiveness of RTA is higher in GMFCS level III and IV children than in level I and II children. Our data suggested that the lower the baseline functional skills of the patient, the more significant the effect of RTA on GMFM total score and each GMFM dimension. Our results showed that improvement in less complex functions (dimension A) was inversely associated with GMFCS level. The effect of age was observed only in GMFM dimension D, where the improvement in standing was greater in the older children.

Previously reported data and results were not consistent in: participant profile (often individuals in different GMFCS levels were considered as one group) [26,28,31], intervention applied (exoskeleton, lokomat, and treadmills, sometimes combined with conventional physical therapy) [12,14,17,24], and the number of treatment sessions (from 3 to 20) [12,25,26]. The primary goal of our protocol focused on improving gait training. Therapy was based on gait (with exoskeleton and treadmill) and balance training (on stabilometric and dynamographic platforms). The therapy program was very intensive, and consisted of 30 sessions within two months. Participants were considered as one group and then were divided into groups based on ambulatory level. Each participant completed all the examinations and collected data. The complexity of our data and the therapy schedule provide clear information about which individuals may benefit the most from RAT and what changes could be expected. 

### Limitations

The main limitation of the study was the validity of the data from the clinical and functional examinations. Our outcome measures were data from clinical examinations of range-of-motion (collected with a manual goniometer), spasticity, selective motor control, muscles strength, and functional assessment as evaluated by GMFM. Although those are well-established and highly recommended assessment tools, the quality of the data may be of concern. To reduce errors and improve data quality, examiners were limited to two well-trained and highly experienced physical therapists. Because of the difficulty of recruiting study subjects in the clinical setting, a control group was not used in this study. Further studies should include a matched control group, which follows the same duration and intensity of therapy as an objective comparison to the target population. Although the sample size of the present study (*N* = 28) is higher than previous similar studies, the authors are aware of the insufficient number of study subjects. To confirm our findings, the results of the present study need to be further verified with full-scaled studies in the future. 

RAT was developed to provide task-specific and goal-focused therapy for patients with motor impairments. The perfect therapy scheme, patient profile, and dose are still not clear, and the outcomes reported after therapy are not consistent. The results of the present study suggested that integrating RAT into rehabilitation programs can be beneficial to ambulatory individuals with CP. However, the optimal dose, schedule, and patient profile should be further determined in future studies.

## 5. Conclusions

The present study suggests that the RAT program with the described protocol may improve the general gross motor functions of individuals with CP in GMFCS level I and II, and can improve primarily performance on less complex GMFM items for items in GMFCS levels III and IV. Our results also suggest that a lower baseline functional level is related to a greater functional improvement. Older individuals were noticed to improve more in GMFM dimension D. Regarding impairments evaluated by clinical examinations, no change was noticed in the studied individuals after RAT program intervention. Moreover, the strength of knee muscles was not affected.

## Figures and Tables

**Table 1:** RAT therapeutic schedule for RAT.

**10 Min on Gamma VAST (AC International East, Knurów, Poland):**
Analysis of load distribution between left and right sides of the bodyside (for balance training).
Create individual training difficulty by the real-time biofeedback.
**5-Min break**
**10 Min on Alfa VAST (AC International East, Knurów, Poland):**
Dynamic analysis of center of pressure displacement.
Training of balance according to the amount of displacement.
**10-Min break**
**45 Min of EksoGT (Ekso Bionics, Richmond, CA, USA):**
Analysis of required support of each lower limb joint.
Customized gait training with different levels of support.
The training starts from a shorter period (10 to 15 min). Depending on endurance, the usual walking time range from 30 min to 1 h.
**15-Min break**
**2 × 15 Min on Zebris THQ-M-3i Treadmill (zebris Medical GmbH, Isny im Allgäu, Germany):**
Analysis of spatiotemporal gait parameters and endurance.
Virtual reality training for walking balance and gait.
**5-Min Break**
The whole therapy program was performed under the supervision of two physical therapists experienced with RAT.

**Table 2:** Changes in functional assessment between visits (t1: before therapy; t2: after therapy).

	All Groups							Group I (GMFCS = I and II)							Group II (GMFCS = III and IV)						
	t_1_			t_2_			*p*	t_1_			t_2_			*p*	t_1_			t_2_			*p*
	Min [%]	Max [%]	Mean [%]	Min [%]	Max [%]	Mean [%]		Min [%]	Max [%]	Mean [%]	Min [%]	Max [%]	Mean [%]		Min [%]	Max [%]	Mean [%]	Min [%]	Max [%]	Mean [%]	
GMFM	19.93	99.44	99.44	21.63	21.63	21.63	<0.01	65.21	65.21	65.21	65.59	65.59	65.59	<0.01	19.63	19.63	19.63	21.56	21.56	21.56	<0.01
GMFM part A	76.47	100.00	100.00	80.39	80.39	80.39	<0.01	92.15	92.15	92.15	96.07	96.07	96.07	0.04	76.47	76.47	76.47	80.39	80.39	80.39	0.03
GMFM part B	21.67	100.00	100.00	20.00	20.00	20.00	0.02	85.00	85.00	85.00	96.66	96.66	96.66	0.03	21.67	21.67	21.67	20.00	20.00	20.00	0.26
GMFM part C	0.00	100.00	100.00	7.14	7.14	7.14	<0.01	14.28	14.28	14.28	14.28	14.28	14.28	0.02	0.00	0.00	0.00	7.14	7.14	7.14	0.01
GMFM part D	0.00	100.00	100.00	0.00	0.00	0.00	<0.01	61.54	61.54	61.54	61.54	61.54	61.54	<0.01	0.00	0.00	0.00	0.00	0.00	0.00	0.13
GMFM part E	0.00	97.22	97.22	0.00	0.00	0.00	<0.01	19.44	19.44	19.44	54.17	54.17	54.17	<0.01	0.00	0.00	0.00	0.00	0.00	0.00	0.08

**Table 3:** Changes in clinical assessment between visits (t1: before therapy; t2: after therapy).

Parameter	t_1_			t_2_			*p*
	Minimum [°]	Maximum [°]	Mean [°]	Minimum [°]	Maximum [°]	Mean [°]	
Hip flexion	80	80	80	85	85	85	0.53
Hip abduction	15	15	15	20	20	20	0.50
Hip internal rotation	10	10	10	25	25	25	0.18
Hip external rotation	0	0	0	0	0	0	0.87
Hip anteversion angle	15	15	15	10	10	10	0.54
Knee extension	−10	−10	−10	−15	−15	−15	0.76
Knee flexion	100	100	100	100	100	100	0.05
Unilateral popliteal angle	30	30	30	30	30	30	0.21
Bilateral popliteal angle	25	25	25	20	20	20	<0.01
Ankle dorsiflexion (knee flexion = 0°)	−35	−35	−35	−40	−40	−40	0.56
Ankle dorsiflexion (knee flexion = 90°)	−10	−10	−10	−20	−20	−20	0.76
Ankle plantarflexion	15	15	15	20	20	20	0.17

**Table 4:** Changes in instrumental strength assessment between visits (t1: before RAT; t2: after RAT).

Parameter	t_1_			t_2_			*p*
	Minimum (Nm)	Maximum (Nm)	Mean (Nm)	Minimum (Nm)	Maximum (Nm)	Mean (Nm)	
Knee extension	5.5	5.5	5.5	8.8	8.8	8.8	0.47
Knee flexion	0.1	0.1	0.1	0.0	0.0	0.0	0.05

## Data Availability

The data that support the findings of this study are available on request from the corresponding author. The data are not publicly available due to privacy or ethical restrictions.
